# Directivity Improved Antenna with a Planar Dielectric Lens for Reducing the Physical Size of the On-Vehicle Communication System

**DOI:** 10.3390/s24216831

**Published:** 2024-10-24

**Authors:** Seongbu Seo, Woogon Kim, Hongsik Park, Yejune Seo, Dohyun Park, Hyoungjong Kim, Kwonhee Lee, Hosub Lee, Sungtek Kahng

**Affiliations:** 1 Department of Information & Telecommunication Engineering, Incheon National University, Incheon 22012, Republic of Korea; castlerich@inu.ac.kr (S.S.); wgon1002@inu.ac.kr (W.K.); p0306ok@inu.ac.kr (H.P.); 2 NS-Satellite RTDC ITR Center, Incheon National University, Incheon 22012, Republic of Korea; m.june@inu.ac.kr; 3 LIGNEX1, Bundang-gu, Seongnam-si 13488, Republic of Korea; dohyun.park@lignex1.com (D.P.); hyoungjong.kim2@lignex1.com (H.K.); kwonhee.lee@lignex1.com (K.L.); hosub.lee@lignex1.com (H.L.)

**Keywords:** array antenna, satellite, signal strength, radio sensor, metamaterial surface, transmission and reflection

## Abstract

As the physical size of a communication system for satellites or unmanned aerial vehicles demands to be reduced, a compact antenna with high directivity is proposed as a core element essential to the wireless device. Instead of using a horn or an array antenna, a unit planar antenna is combined with a surface-modulated lens to convert a low antenna gain to a high antenna gain. The lens is not a metal-patterned PCB but is dielectric, which is neither curved nor very wide. This palm-sized lens comprises pixels with different heights from the backside of PolyPhenylene Sulfide (PPS) as the dielectric base. The antenna gain from the unit antenna of 4.5 cm × 4.5 cm is enhanced by 10 dB with the help of a compact dielectric lens of 7.5 cm × 7.5 cm at 24.5 GHz as the frequency of interest. The antenna design is verified by far-field measurement as well as near-field observation, including sensing a metal object behind a blocking wall by using an RF test bench. Moreover, antenna performance is understood by making a comparison with conventional designs of antennas in terms of directivity and physical sizes.

## 1. Introduction

Connecting one node to another on a communication network wirelessly is not a surprise any longer. As the level of the technology has advanced to suit user requirements by introducing more complicated architectures, algorithms and circuitry than before, and some elements are excluded from the system and new elements are brought in, there are elements that are indispensable to the wireless communication system. One of them is frequency. Working as the primary element to form a channel between a transmitting node and a receiving node, the frequency of one node should be tuned to that of another. The frequency has been driven to go up through GHz-mobile services like UMTS, WiFi and LTE-A to X-band, Ku-band and higher. Increasing frequency comes from the goal of securing a high data transmission rate and targeting a small number of receivers instead of many distributed over a wide angle as elaborated in [1,2,3]. Real-time interaction added video streaming and inter-satellite operation in an LEO constellation, which needs much faster communication than that. For this purpose, frequency is set at Ku-band above 12 GHz or K-band above 18 GHz. It is suggested to exceed 20 GHz for broader bands, and when it is near 30 GHz, they call it the millimeter-wave band. This takes advantage of the increased data transfer rate, but on the flip side, the signal is sensitive to the environment and ends up with path loss.

Wireless device developers have built systems working in the high frequency band by employing high-gain antennas; they look for solutions to compensate for path loss like array antennas [4]. When it comes to high-gain antennas, a horn or the parabolic reflector can usually be considered. However, these volumetric structures take up a relatively large space and make the whole system heavy. Hence, the microstrip patch array antenna is preferred as a planar structure [5,6]. Whether military or commercial, use-cases demanding increased antenna gain see the array antennas placed in the transceiver. Nonetheless, when entering the millimeter-wave band, drawbacks of the array antenna are addressed. The RF signal undergoes an increasing amount of loss as it travels from the input port to the far-end in the power divider as a result of high sensitivity to the loss factors of the dielectric and conductor. When the array antenna should be larger to catch up with a necessary gain against attenuation along the signal path, the growth in loss overshadows the antenna gain. This causes heating from beamforming chipsets.

Given that the use of patch array antennas runs into such problems, a transmitarray or superstrate is worthy of being tried to tackle them. Placed above the antenna as the source of radiation, the superstrate is introduced to enhance the strength of far-field radiation, and it is also called a metasurface lens, which is analogous to lenses in optics.

**Figure 1:**
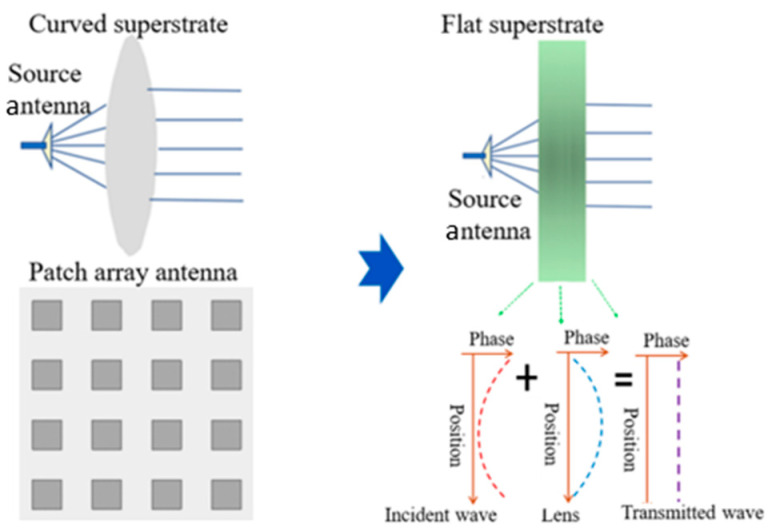
The array antenna or curved lens conventionally used in high-frequency band communication can be replaced by a metasurface lens adjusting the phase of the transmitted wave.

A diverging wave from the source antenna can be changed to high directivity at the far-zone field by way of a superstrate as described in Figure 1. A conventional lens could generate phase shifts for the incoming rays by bending the surfaces of the dielectric material. As the gap between the source antenna and the superstrate needs to be shorter than the conventional technique where the plane of the incidence is far enough from the source antenna, the phase distribution of the superstrate is implemented by using the idea of new materials. There are representative methods such as the phase compensation approach, graded index approach, double negative parameters, etc. Some examples of them are briefly mentioned. A multi-layered PCB as the superstrate was added to the source antenna by Datthanasombat et al. to change the quasi-spherical wavefronts into parallel wavefronts [7]. Raising the frequency to a higher channel in the mm wave band, a Q-band transmitarray was presented by Kaouach et al. using a layered PCB for increasing the gain [8]. Dussoptia et al. took a similar step to the previous references to have a high antenna gain over an expanded bandwidth [9,10]. Ray optics were employed at the initial stage of the design by Bai et al. to make the radiation aperture wider for increasing directivity [11]. Wang and Liang had more layers in the superstrate with gaps and trimmed the gaps and heights there [12,13]. Slits were introduced to the metal plane to couple the fields from the source with the top layer [14]. Their geometries could generate beams of improved directivity.

In this paper, different from the PCB-type superstrates that conventional methods use, a dielectric slab is employed as a small footprint lens for the source of radiation. As pictorially explained below, the proposed structure gives wireless systems of aerospace communication positive effects.

**Figure 2:**
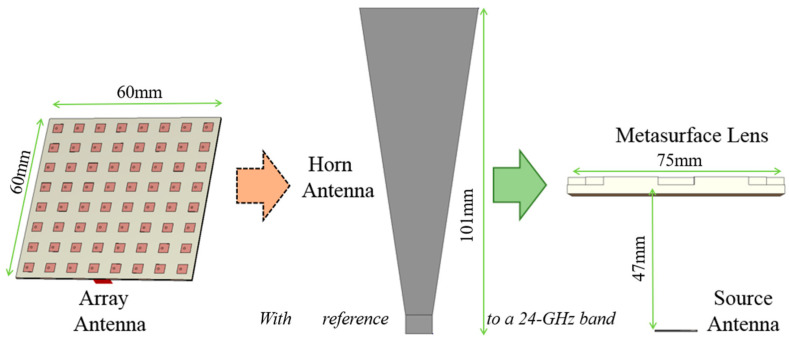
The proposed antenna compared to a horn and feed-lossy array in view of the size.

Aircraft and satellites located at high altitudes can communicate with ground stations on which parabolic reflectors as well as horn antennas are mounted. The horn antenna has a reputation for directional radiated fields. Its antenna gain is such a merit that alone or combined with a reflector, it can generate high directivity as much as a large patch array antenna and relieve the active RF block of necessitating multiple-stage power amplifiers. As is shown in Figure 2, the horn antenna is long while the other antennas of the same antenna gain are relatively short. This metallic waveguide antenna can be replaced by the proposed antenna. As is given on the right side of Figure 2, a dielectric slab is placed over the source antenna. As conventional metasurface lenses take the form of multi-layered and wide PCBs, there are air gaps or bonding films between one wide layer and another. On the contrary, the contribution is given by making a flat lens out of one homogeneous dielectric slab PPS, which is better than multi-layered PCB geometry in terms of errors from vertical and horizontal misalignment of stacking and uncertainty of materials and thickness of adhesive films. These errors will make the electromagnetic properties of the antenna of interest worse in K- or Ka-band and above. Therefore, a single layer material is good for the lens, but for it to work up to expectations, it requires a sufficient thickness that a single PCB substrate in the market cannot satisfy. This is why PPS is chosen. In order to make the single dielectric slab play the role of lensing, the required phase distribution of the structure is implemented with certain heights in pixels. Drilling and grinding in the fabrication process are adopted to make the surface of the lens. This increases the antenna gain by around 10 dB with a lens of 7.5 cm × 7.5 cm at 24.5 GHz, with reference to the source antenna. The design is validated by far-field and near-field tests. Also, it is proven that the lens can improve the function of sensing a metal object behind a blocking wall. This is tested by using an RF test bench comprising a two-port vector network.

### 1.1. Design of the Source Antenna

The proposed structure comprises two parts. One is the source antenna and the other is the lens. Firstly, the design of the source antenna is addressed. While most of the literature on planar antennas have a horn antenna or a single patch antenna as the primary source, we do not follow suit. When a horn antenna is used to excite the superstrate, the gap between the two parts is enormous. The superstrate becomes very large as the source antenna is located far away, which is improper for the objective of size reduction. As for a single patch antenna, since the initial gain is small, to eventually have a large antenna gain, it is unavoidable to make the geometry large.

**Figure 3:**
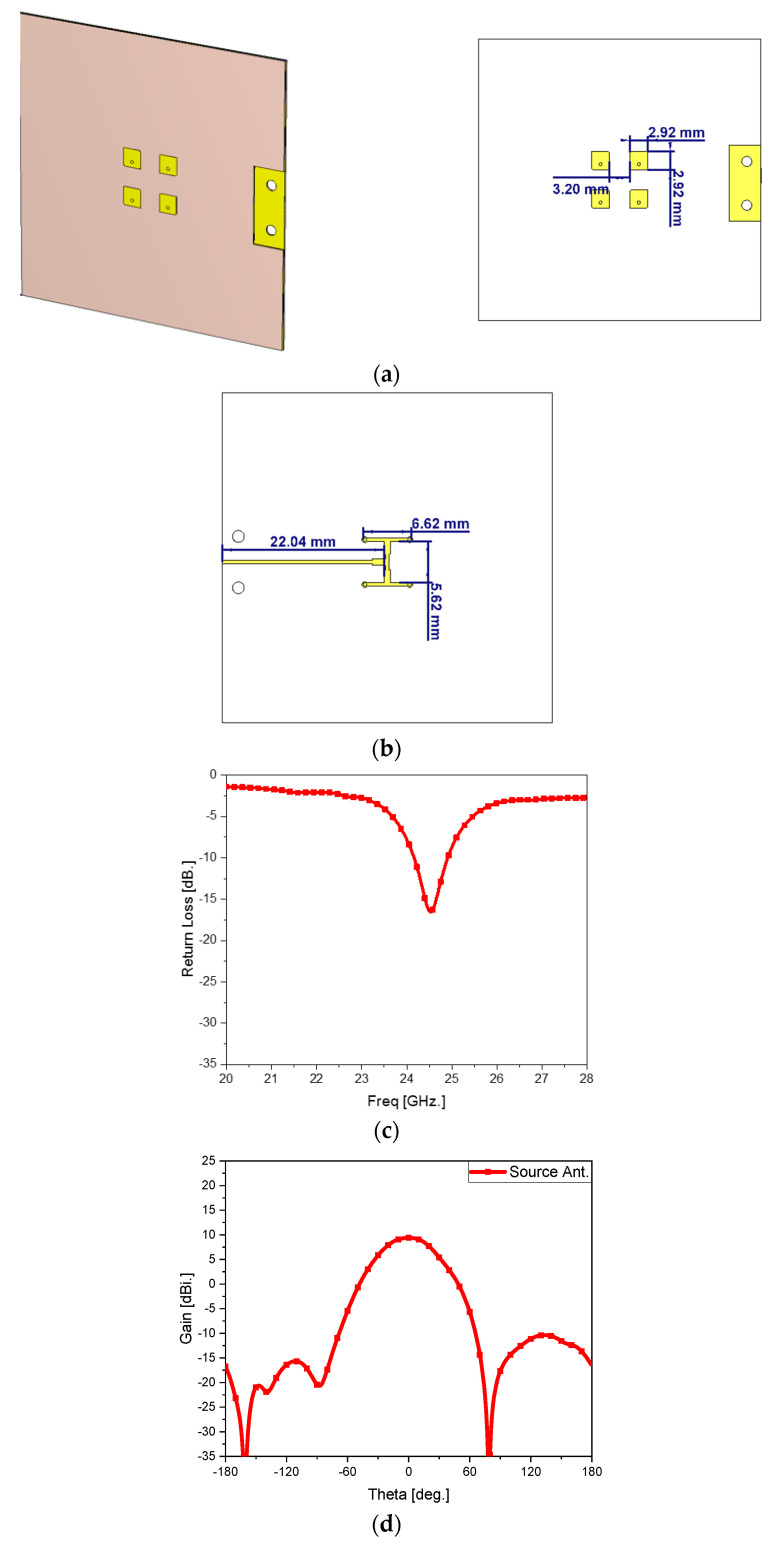
The source antenna: (**a**) 3D view and top view, (**b**) bottom view, (**c**) reflection coefficient, and (**d**) beam pattern.

The source antenna is a 2 × 2 array as in Figure 3a,b. Generating an antenna gain higher than a single patch helps it achieve high directivity in the final structure. Each patch has an area of 2.9 mm × 2.9 mm on a 45 mm × 45 mm substrate of 4350B. The reflection coefficient in Figure 3c,d shows the operating frequency as desired and the beam pattern with an antenna gain of 9 dBi. Next, it is combined with the lens.

### 1.2. Design of the Lens

The lens is presented as follows.

**Figure 4:**
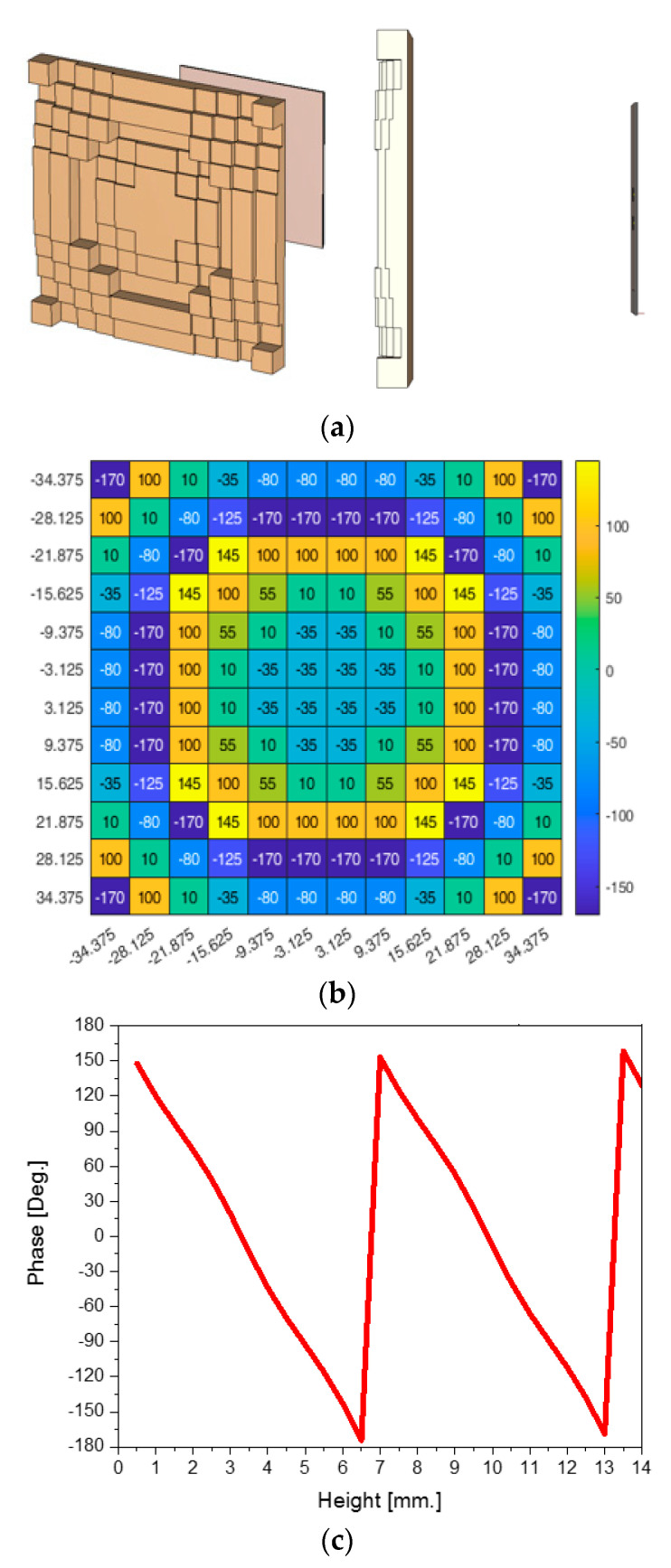

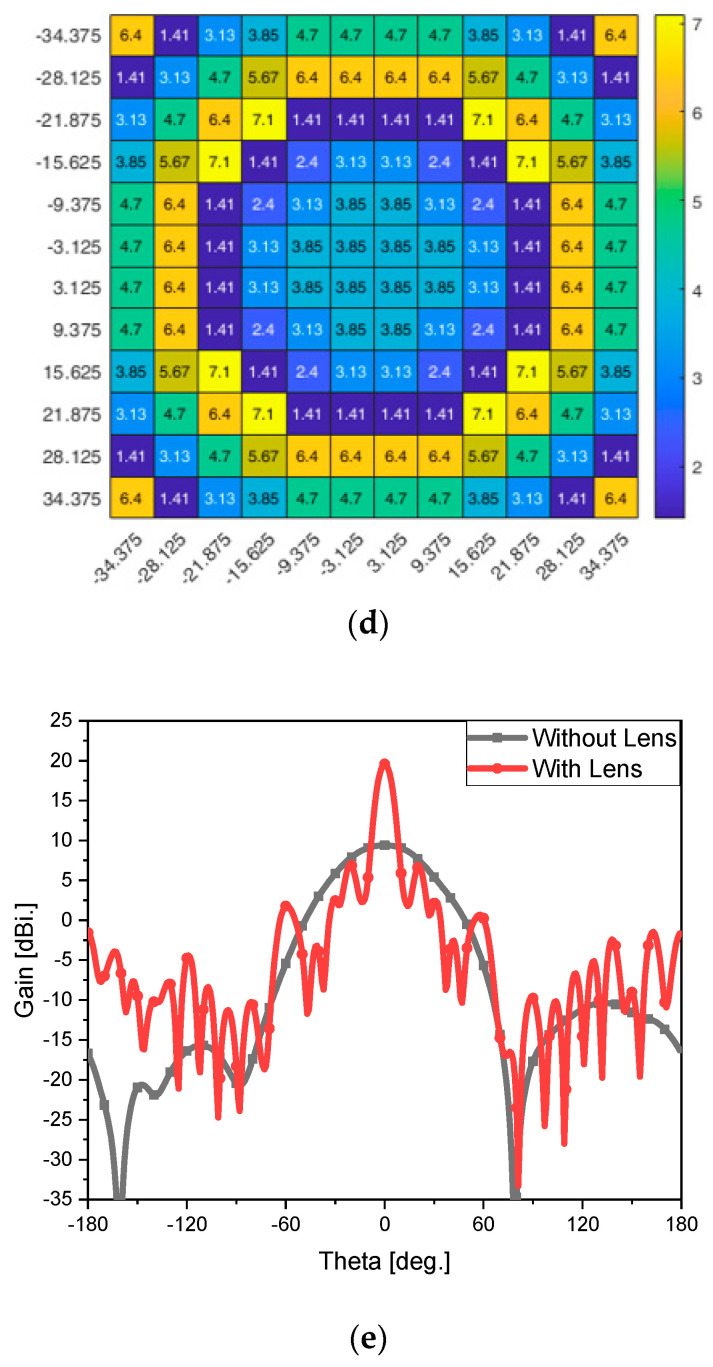
The proposed antenna: (**a**) lens over the source antenna and side view of the proposed structure, (**b**) phase distribution of the lens, (**c**) height of the pixel vs. phase, (**d**) heights of all the pixels of the lens, and (**e**) a comparison of the beam patterns of (**a**) and Figure 3.

A dielectric slab sized 75 mm × 75 mm is placed over the source antenna with a gap of 4.7 cm as given in Figure 4a. On a very compact plane, the surface of the dielectric slab is formed on the basis of the phase distribution of Figure 4b, which is the result of a calculation for converting the phase distribution of the incoming wave from the source to the one required for the beam pointed at the zenith. To implement the phases of the pixels of the surface, which correspond to the tiles of its discretized version, either different permittivity dielectric cubes of the same height or the same permittivity of different heights is possible to think of. Realization begins by relating the height of the design parameter of the dielectric unit with the phase as in Figure 4c. This is applied to expressing the phases with the heights of all the pixels as in Figure 4d working for the incoming wavefront from the 2 × 2 array antenna. When phases of 100° and −170° are required, Figure 4c maps them to 1.41 mm and 6.4 mm, respectively, in height. This mapping applies to other phases and pixels. Through this process which is explained again in Appendix A, the antenna is realized. The simulated far-field patterns of the source antenna alone and the lens-combined antenna are plotted based on this implementation. It is observed that there is a distinct enhancement in the view-point of antenna gain from the source antenna to the lens-added patch antenna. About a 10 dB increment is noted by looking at Figure 4e. It seems equivalent to the effect of a 10 cm long horn antenna in that it can generate a far-field pattern of almost the same gain as the proposed structure.

## 2. Characterizing the Antennas Through RF Measurement

The proposed design scheme to substantially enhance the antenna gain of a small-sized array antenna is validated by fabrication of the prototypes and experiments with them. It has procedural steps from the primary source antenna to the lens-combined geometry. As steps 1 and 2, the primary source and its modified version with a fixture are electromagnetically characterized.

**Figure 5:**
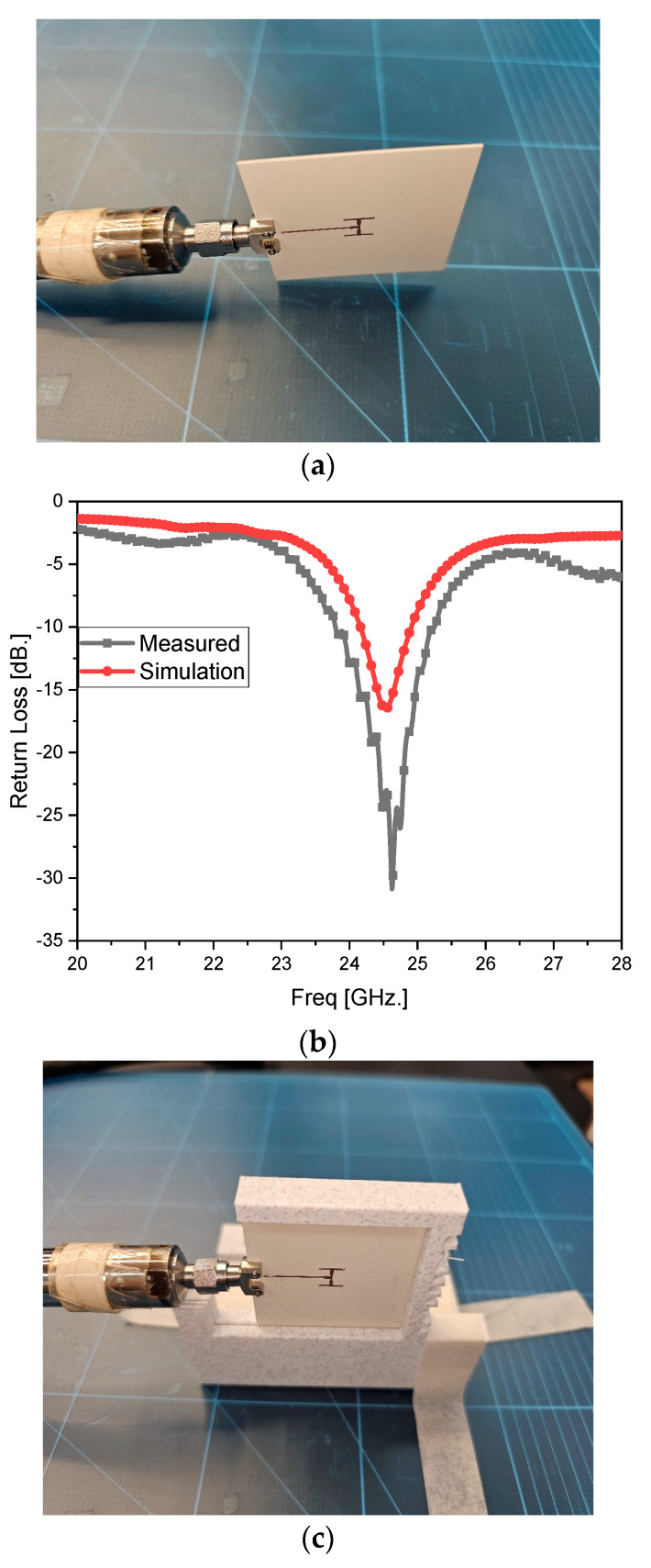

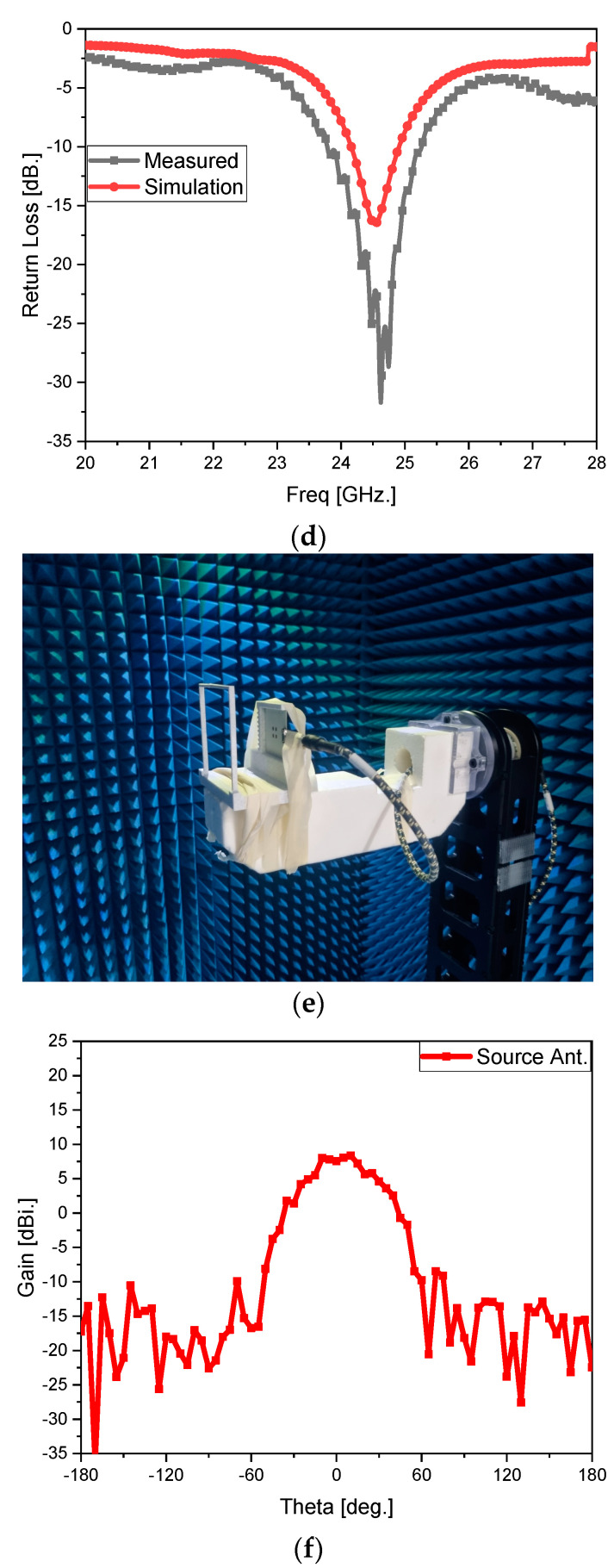
Experimental observation of the performances of the source antenna and the one held by a fixture. (**a**) Prototype of the source antenna. (**b**) Simulated and measured reflection coefficient. (**c**) Prototype of the source antenna harnessed by the jig. (**d**) Simulated and measured reflection coefficient of (**d**,**e**). (**e**) Test setup. (**f**) Measured beam pattern.

In step 1, the electrical property of the source antenna, which is prototyped as in Figure 5a, is checked. The 2 × 2 array’s metal patches on 4350B as the substrate mentioned in Figure 3 are fed by a power divider whose port is connected to the port coming from the vector network analyzer. Figure 5b shows that it meets the resonance condition in that S_11_ as the reflection coefficient at the port becomes lower than −10 dB at 24.5 GHz. The bare board as above is handled in just the initial phase of a series of tests, and should be put into a fixture for experimentally investigating far-field radiation as in step 2. In the antenna test setup, it is necessary to place the antenna on a feeder or turn-table with the fixture, and this harnessing may affect the electrical and electromagnetic properties of the antenna because it is not the material of air but metal or dielectrics. The effect of the fixture on the antenna is considered through modeling and physical implementation as in Figure 5c,d. Using a 3D-printed fixture of PLA, the simulated and measured S_11_ curves agree with each other, showing resonance at the target frequency. Figure 5f of the AUT in the anechoic chamber to (d) presents the simulated and measured beam patterns as the same as Figure 3d as a broad beam.

**Figure 6:**
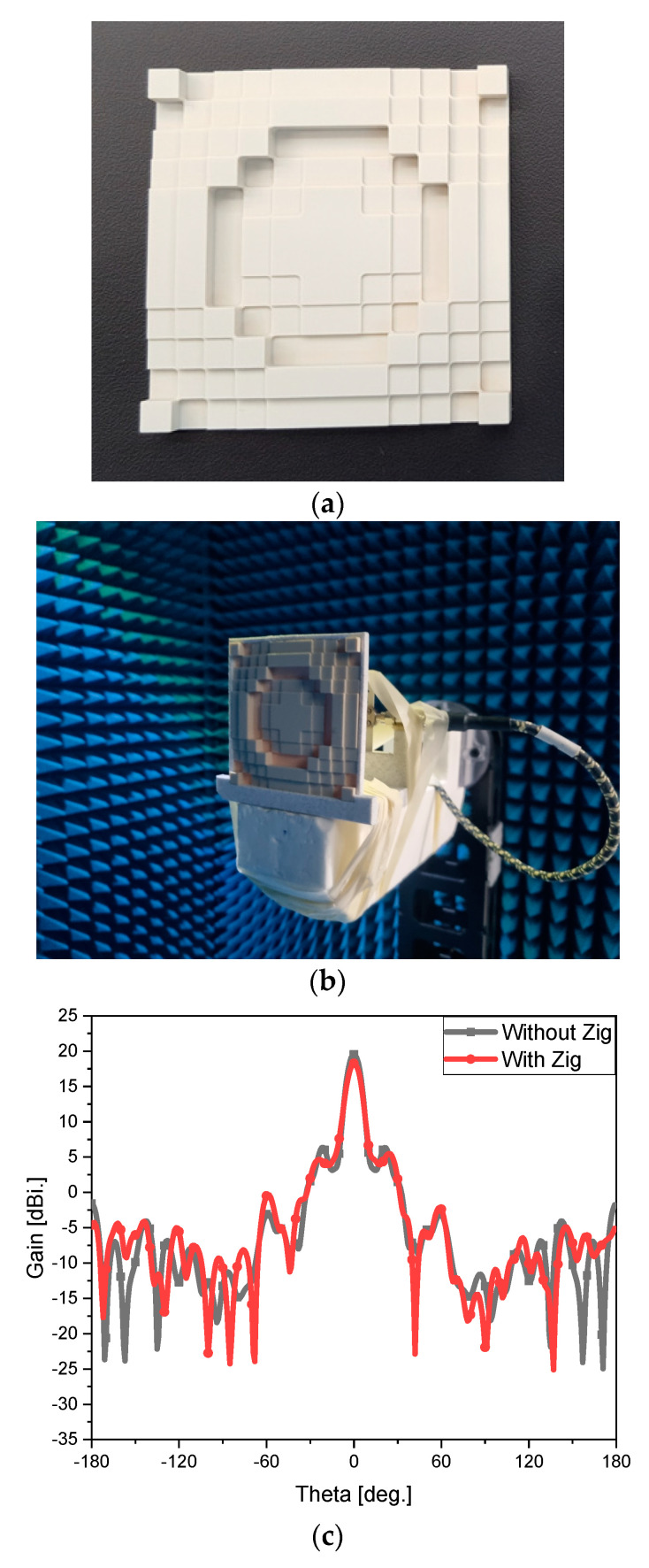

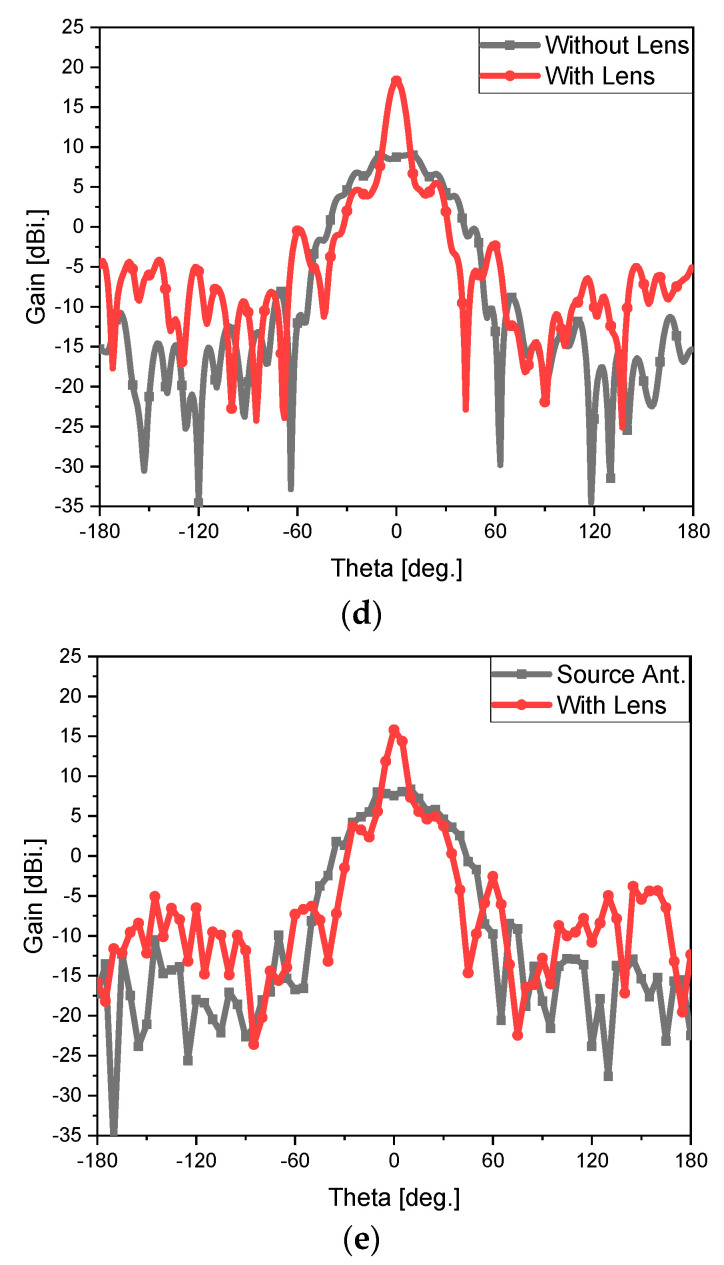
The simulated and measured performance of the fabricated lens antenna. (**a**) Fabricated lens antenna. (**b**) Test setup. (**c**) Simulated far-field patterns without and with the jig. (**d**) Simulated far-field patterns without and with the lens and the increased gain. (**e**) Measured far-field patterns without and with the lens and the increased gain.

The lens is manufactured as in Figure 6a, which is a homogenous dielectric slab. The model in Figure 6b implies that the lens goes into the unoccupied area of the jig of Figure 5c. This model is simulated, and its far-field pattern is obtained. Figure 6c plots the simulated beam patterns of the antenna without and with the jig, and they have no difference except for roughly 1-dB loss as in 19 dBi to 18 dBi. Measurement that verifies the design and simulation like Figure 6d is conducted by employing a full setup of Figure 6e. The objective of the proposed method is confirmed by observing the remarkably enhanced gain as in Figure 6e though both the beam patterns experience loss due to loss of the cable, its torsion, uncertainty from mechanical contacts, etc. [15,16,17,18,19].

## 3. Observation of the Strengths of the Lens in View of Wireless Sensing

So far, the proposed structure has been dealt with primarily from the standpoint of antenna functions and geometrical characteristics. Meanwhile, secondary aspects of the antenna such as playing a core role in wireless sensing are addressed. The line of sight (LoS) link, as in free-space communication, is dubbed into reflection by a metal plane. In addition, the antenna is used to detect hidden objects as in radars.

**Figure 7:**
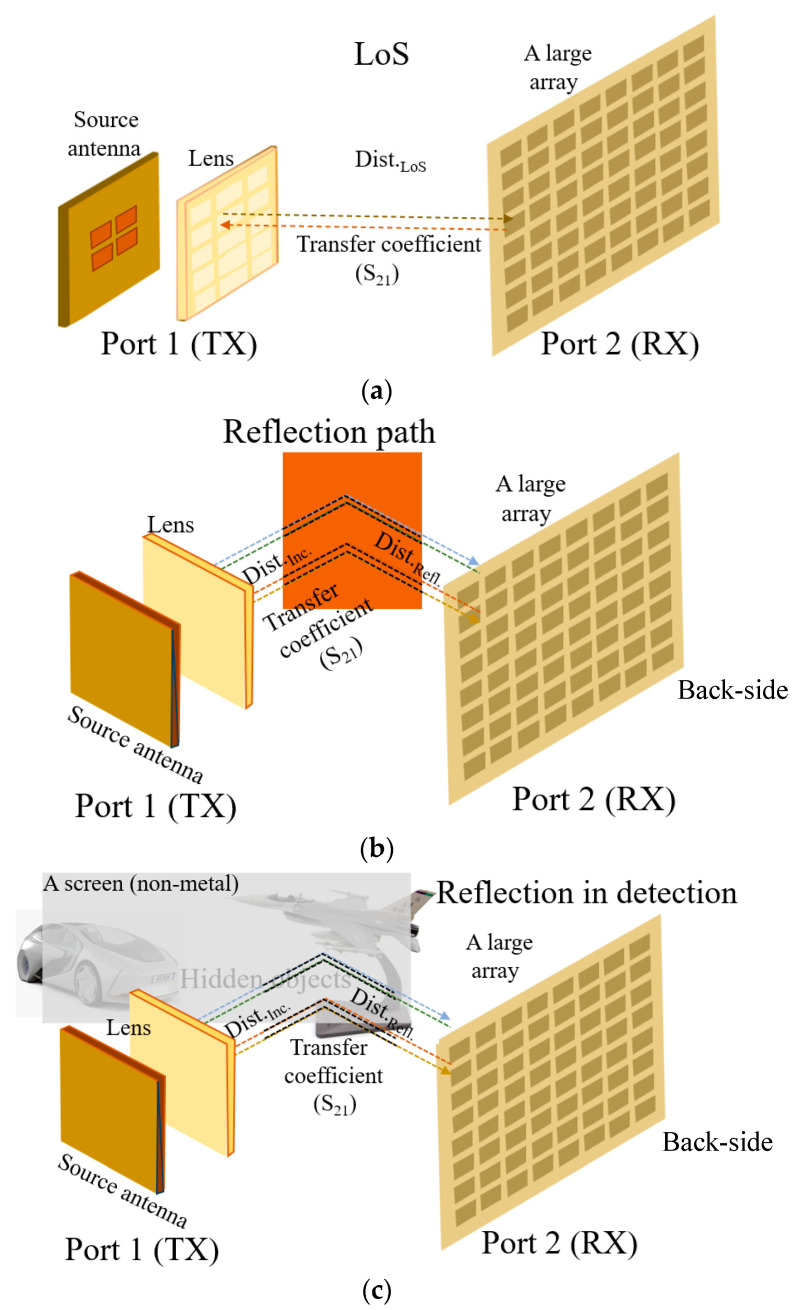
Schemes of testing various links between the TX and RX. (**a**) Sensing the signal in the LoS. (**b**) Sensing the signal reflected by the metal plane. (**c**) Sensing objects hidden by a screen (foam).

Far-field measurement showing the EM power distribution on the spherical space at a long distance from the AUTs requires relatively high input power. With low input power, near-zone experiments using the VNA with two ports can characterize the AUTs. Figure 7b,c show the reflected signals. They have 50 cm as the traveling distance. Port 2 adopts an 8 × 8 array to amplify the signal strength in an RF-passive manner, considering the low power.

**Figure 8:**
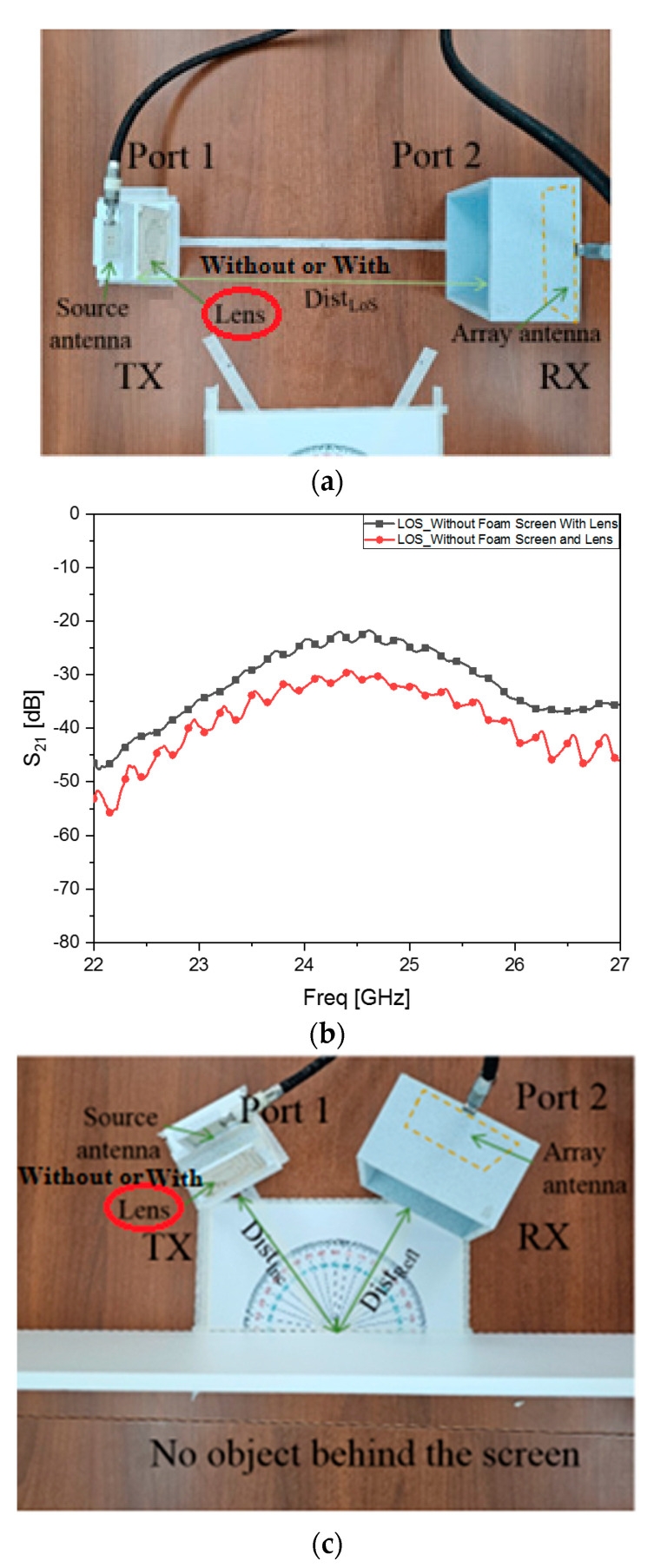

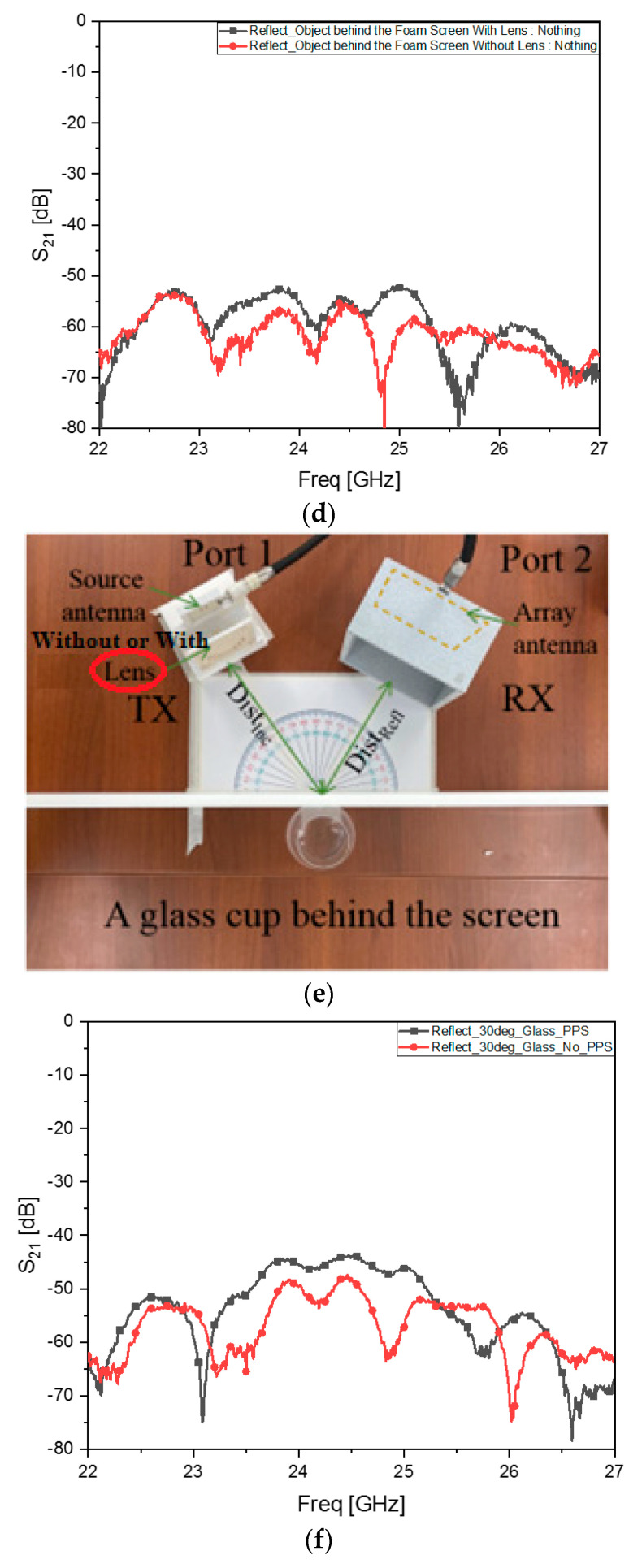

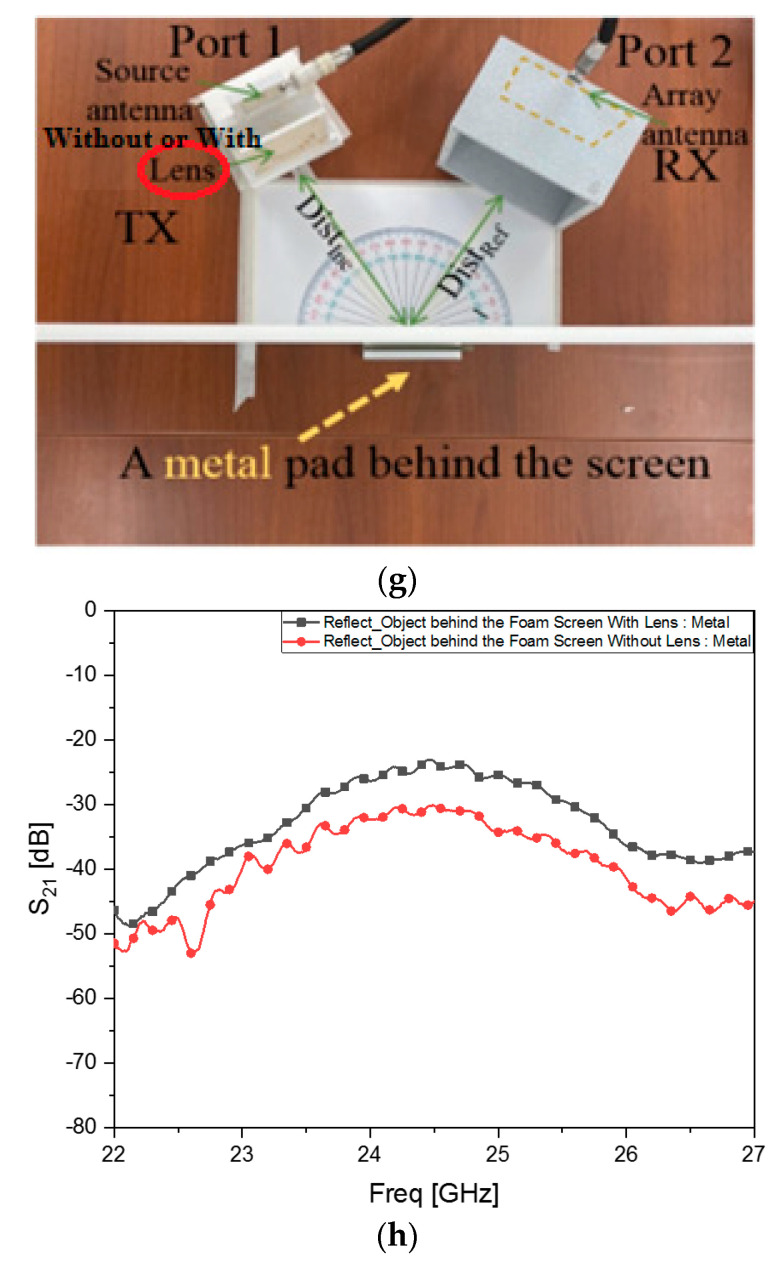
Testing various links between the TX and RX including RF sensing. (**a**) Sensing the signal in the LoS without or with the lens. (**b**) S_21_ transmission coefficients of the LoS signals without and with the lens. (**c**) Without or with the lens, sensing the signal reflected by no object behind the screen. (**d**) S_21_ transmission coefficients of the reflected signals without and with the lens while having no object behind the screen. (**e**) Without or with the lens, sensing the signal reflected by a glass cup behind the screen. (**f**) S_21_ transmission coefficients of the reflected signals without and with the lens while having a glass cup behind the screen. (**g**) Without or with the flat lens, sensing the signal reflected by a metal pad behind the screen. (**h**) S_21_ transmission coefficients of the reflected signals without and with the lens while having a metal pad behind the screen.

Hands-on experiences of wireless links from the TX to the RX are acquired through checking the transmission coefficient from one antenna at port 1 to that at port 2 of the VNA. As mentioned previously, contrary to the far-field pattern tests, since the VNA has a limited level of input power, the test setups in Figure 8 take 50 cm as the distance for both the straight and reflected paths. At first sight, the distance might look very short, but it amounts to 40.8 wavelengths, which seems like quasi-far-field and makes the millimeter-wave signal become very weak at the RX. In order to increase the sensitivity of the signal reception, instead of a simple antenna, an 8 × 8 array is connected to the RX side. On the TX side, the tests take turns from the source antenna to the flat lens-combined structure. The greater antenna gains on both sides lead to sensible results.

(1) $$P_{RF\_RX}{|}_{{Ant.}_{Ref}}=\zeta \times \frac{{{\left|E_{0}\right|}_{Freq.}}^{2}}{{{Dist.}_{Ref}}^{2}}\times G_{TX\_Ref}\times G_{RX\_Ref}\times \zeta_{RX}$$

(2) $$P_{RF\_RX}{|}_{{Ant.}_{New}}=\zeta \times \frac{{{\left|E_{0}\right|}_{Freq.}}^{2}}{{{Dist.}_{Ref}}^{2}}\times G_{TX\_New}\times G_{RX\_New}\times \zeta_{RX}$$

(3) $${\Delta P}_{RF\_RX}\left[dB\right]=P_{RF\_RX}{|}_{Ant._{New}}\left[dB\right]-P_{RF\_RX}{|}_{{Ant.}_{Ref}}[dB]$$

where *P_RF_RX_*, ${{\left|E_{0}\right|}_{Freq.}}^{2}$, *G* and ζ imply the RF power of the received signal expressed in S_21_, initial RF power, antenna gain and coefficient of the electromagnetic radiated power, respectively, at the frequency of interest. These findings are verified through the following tests. Firstly, LoS signals are observed as S_21_ regarding the source antenna as the TX or the proposed lens as the TX as in Figure 8a. Between the source antenna and the 8 × 8 array, S_21_ is −31 dB, which jumps to −23 dB as in Figure 8b by substituting the flat lens for the source antenna. Comparing the values of S_21_ at the target frequency, −30 dB without the lens becomes −21 dB, meaning an improvement in the transmission. The direct path is changed to the V-shaped reflected path to investigate the capability of RF sensing on a target placed behind a screen. Figure 8c is the photograph of the reflected paths for two cases (without or with the lens), which has nothing behind the foam screen in common. Foam is reckoned to be a type of air for permittivity. Since there is no reflection due to nothing being hidden, the port receives −55 dB as in Figure 8d, which is understood as empty. The curves are compared and show both of them having no reflected signal of a measurable value. Figure 8e involves the sensing of a glass cup behind the screen with the proposed antenna. Seeing Figure 8f, S_21_ increases to −47 dB from the cases that hide nothing. The lens results in −44 dB. Compared to the case without lens, the signal level becomes larger by 5 dB; when referring to the LoS case, the increment rate is lower due to wave scattering by the round shape and material loss of the cup. Lastly, a palm-sized metal pad is introduced to the backside of the screen as in Figure 8g. The object is made out of copper. Figure 8h presents that the S_21_ values become −24 dB and −31 dB, respectively, for the source antenna alone and the flat lens-combined geometry. An improvement in object sensing is about 10 dB from −31 dB to −22 dB. This effect is compared to the LoS cases, and reflection from the metal has a similar behavior to the direct path. The proposed structure enables the beam to be narrow enough to sense a metal object, which is a few centimeters in width and strong enough to make the reflected signal strengths similar to LoS cases.

## 4. Conclusions

An antenna comes to have a notably increased antenna gain by making a contribution of developing a compact and flat lens for the usage of 24.5 GHz, representing millimeter-wave bands, and necessitating solutions to compensate for fast attenuation in propagation. The proposed lens is a 75 mm × 75 mm sized homogeneous dielectric slab; the surface of which has different heights on the pixels corresponding to the desired phases for the purpose of changing the ones of the incident wave to those of the transmitted plane wave. As a superstrate whose footprint is not large over the 2 × 2 array as the source antenna, the flat lens enhances the antenna gain of the source antenna by about 10 dB. This effect from this contribution is equivalent to what a 10 cm long horn or 8 × 8 patch array can generate, which means effective miniaturization is obtained. The proposed design is validated by prototyping and far-field measurement. In addition, near-zone signal strength observation using the antennas fed by ports of the VNA as the test bench has been conducted to figure out advantages in the lens-combined structure. The signal strengths are measured on the straight and bouncing paths between the source antenna alone or planar lens-combined geometry at port 1 and the 8 × 8 array for sensitivity increment at port 2. When the TX employs the lens-combined antenna, the signal strength improves by nearly 9 dB. Additionally, this improvement helps sensing of the hidden object and has a better performance in that the level of detection of a small metal object goes up by almost 9 dB. The proposed design can solve the problems of conventional antennas mounted on a satellite payload like patch arrays and horns or parabolic reflector antennas, which suffer from conductor and dielectric loss and large volumes, respectively. In addition, this will be potentially used as a lighter and smaller device in communication and sensing as well.

## Figures and Tables

**Figure A1:**
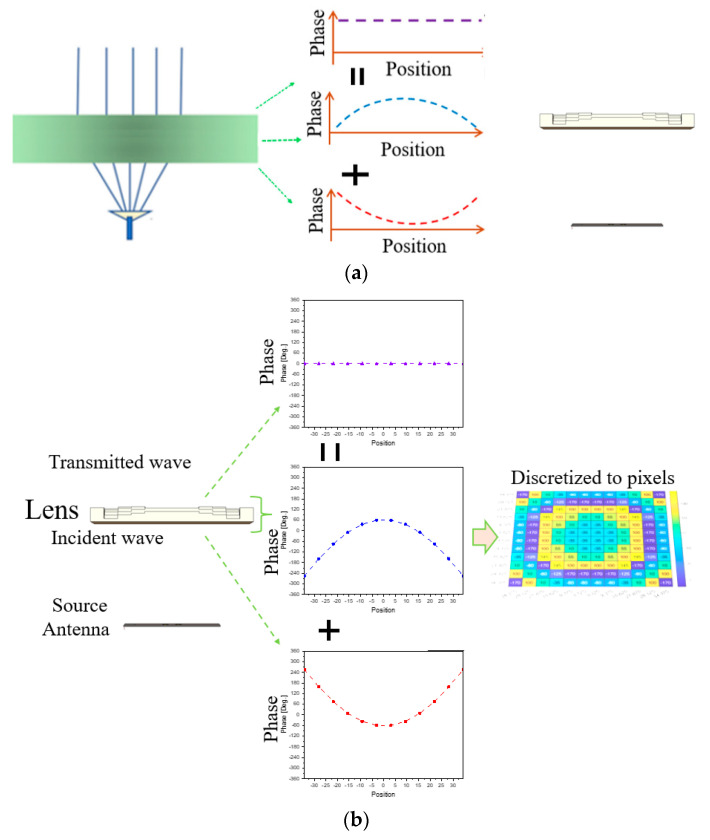
Working principle of the lens. (**a**) Ideal case of phase compensation vs. the real structure, and (**b**) change in the phase from the incident to transmitted waves by the proposed lens.

**Figure A2:**
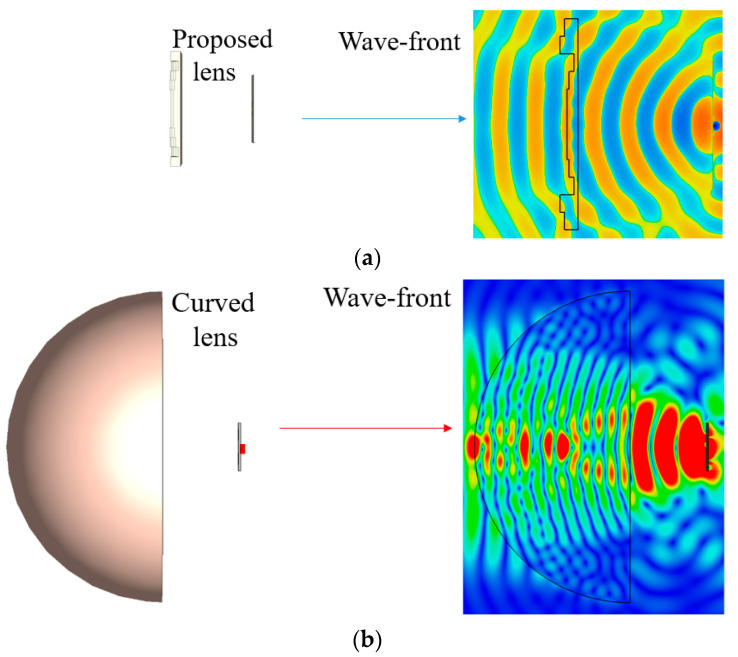
Comparing the flat lens with the curved lens. (**a**) Geometry and the wavefront. (**b**) Structure and the wavefront of the scattered wave.

## Data Availability

Data are contained within the article.

## Appendix A

The geometry and functions of the proposed antenna have been explained step by step. Most of the time, communication system developers tend to adopt patch array antennas, since they are very common for the purpose of producing high directivity and giving the benefit of a low profile. However, the operating frequency of the patch array increases, and the feed circuit comprising the laminated dielectric materials and copper lines becomes lossy and makes the antenna performance very inefficient. Others use horn antennas, which are made out of metal and much less lossy than the patch array. Horn antennas give high antenna gains, but they occupy a large volume. This makes the payload of the satellite heavy. As an alternative method, a lens antenna is suggested. The dielectric is not laminated but has homogeneity. It is flat and able to concentrate electromagnetic flux to generate a high gain. The working principle is as follows.

In Figure A1a, the ideal lens is divided into the three parts. They are the wave from the source antenna, the planar lens and the outgoing wave. The proposed antenna is a flat dielectric slab, which comprises pixels for discretization to adjust the phase of the incident wave to the constant phase distribution on the plane where the transmitted wave is launched as in Figure A1b. The blue curve of the lens is added to the red curve of the incident wave to become that of high antenna directivity.

The phase-compensating lens proposed in this paper can be differentiated from other structures in the references. When comparing the characteristics of the proposed antenna as in Table A1 from others, other lensing structures are fabricated by a chemical process for the laminated substrate but ours is physically manufactured. Regarding the total volume, the proposed lens occupies a small volume. In order to verify the size-reduction effect of the proposed lens antenna, a typical lens, which has a curved surface, is designed and compared.

**Table A1:** Comparing the features of the proposed antenna with other studies.

	Fabrication Process	Multi-Layer	Total Size (Volume)	mm Wave
[8,9,10]	PCB etching	Yes	≥1000 λ^3^, with horn	Yes
[12,13]	PCB etching	Yes	≥1000 λ^3^, with horn	Yes
[14]	PCB etching	Yes	≥1000 λ^3^, with horn	Yes
Ours	Extrusion, molding	No	145 λ^3^, without horn	Yes

The design of the hemispherical lens is conducted as a comparative study. As in Figure A2, it is usual for the curved lens to have a large volume. Both of the structures have a similar antenna gain of about 20 dBi; the proposed structure is distinguished in terms of size, function and manufacturing cost.

## References

[1] Fu Y., Shan Y., Zhu Q., Hung K., Wu Y., Quek T.Q.S. (2023). A Distributed Microservice-aware Paradigm for 6G: Challenges, Principles, and Research Opportunities. IEEE Netw..

[2] Dong Y., Wang H., Yang Z., Hao N., Zhang C., Yu X. (2024). Cell-free ISAC massive MIMO systems with capacity-constrained fronthaul links. Digit. Signal Process..

[3] Gao Z. (2023). Integrated Sensing and Communication With mmWave Massive MIMO: A Compressed Sampling Perspective. IEEE Trans. Wirel. Commun..

[4] Kraus J.D., Marhefka R. (2002). Antennas for All Applications.

[5] Wheeler H.A. (1965). Simple relations derived fom a phased-array antenna made of an infinite current sheet. IEEE Trans. Antennas Propag..

[6] Mailloux R.J., McIlvenna J.F., Kernweis N. (1981). Microstrip array technology. IEEE Trans. Antennas Propag..

[7] Datthanasombat S., Amaro L.R., Harrell J.A., Spitz S., Perret J. Layered lens antenna. Proceedings of the IEEE Antennas and Propagation Society International Symposium.

[8] Kaouach H., Baili G., Baudoin G. (2016). High-efficiency wideband transmit-array antenna with linear polarization in Q-band. IEEE Trans. Antennas Propag..

[9] Dussopt L., Piazzon L., Lesthievent G., Ferrari P. (2019). A V-band switched-beam linearly-polarized transmit-array antenna for wireless backhaul applications. IEEE Trans. Antennas Propag..

[10] Yang Z.Z., Liang F., Zhao Y.Y.D., Wang B.Z. (2019). Metasurface-based wideband, low-profile, and high-gain antenna. IET Microw. Antennas Propag..

[11] Bai H., Wang G.-M., Wu T. (2019). High-Gain Wideband Metasurface Antenna With Low Profile. IEEE Access.

[12] Wang N., Talbi L., Zeng Q., Xu J. (2018). Wideband Fabry-Perot resonator antenna with electrically thin dielectric superstrates. IEEE Access.

[13] Liang J.-J., Huang G.-L., Zhao J.-N., Gao Z.-J., Yuan T. (2019). Wideband phase-gradient metasurface antenna with focused beams. IEEE Access.

[14] Majumder B., Kandasamy K., Mukherjee J. (2015). Wideband compact directive metasurface enabled pair of slot antennas. Electron. Lett..

[15] Rennings A., Otto S., Mosig J., Caloz C., Wolf I. Extended composite right/left-handed (E-CRLH) metamaterial and its application as quadband quarter-wavelength transmission line. Proceedings of the 2006 Asia-Pacific Microwave Conference.

[16] Jang G., Kahng S. (2011). Compact metamaterial zeroth-order resonator bandpass filter for a UHF band and its stopband improvement by transmission zeros. IET Microw. Antennas Propag..

[17] Seo Y., Lee C., Moon I., Ota K., Omote R., Kahng S. (2021). A Planar Millimeter-Wave Resonator-Array to Sense the Permittivity of COP Film with the 5G Handset Back-Cover. Sensors.

[18] Ataloglou V.G., Egorov G., Kim J., Xu G., Dorrah A.H., Ohadi A., Kim M., Eleftheriades G.V. (2022). Static and Reconfigurable Huygens’ Metasurfaces. IEEE Antennas Propag. Mag..

[19] Szymanski L., Gok G., Grbic A. (2022). Antenna Beamforming With Multiple-Input, Multiplr-Output Metastructures. IEEE Antennas Propag. Mag..

